# Evaluation of a novel biomechanics-informed walking frame, developed through a Knowledge Transfer Partnership between biomechanists and design engineers

**DOI:** 10.1186/s12877-023-04443-7

**Published:** 2023-11-13

**Authors:** Sibylle Brunhilde Thies, Susan Bevan, Matthew Wassall, Blessy Kurissinkal Shajan, Lydia Chowalloor, Laurence Kenney, Dave Howard

**Affiliations:** 1 Centre for Health Sciences Research, School of Health & Society, University of Salford, Brian Blatchford Building Room PO28, Salford, Greater Manchester UK; 2 NRS Healthcare, Coalville, LE67 1UB Leicestershire UK; 3 School of Science, Engineering and Environment, University of Salford, Salford, Greater Manchester UK

**Keywords:** Walking frame, Stability, Usability, Design

## Abstract

**Background:**

Walking aids such as walking frames offer support during walking, yet paradoxically, people who self-report using them remain more likely to fall than people who do not. Lifting of walking frames when crossing door thresholds or when turning has shown to reduce stability, and certain design features drive the need to lift (e.g. small, non-swivelling wheels at the front). To overcome shortfalls in design and provide better stability, biomechanists and industrial engineers engaged in a Knowledge Transfer Partnership to develop a novel walking frame that reduces the need for lifting during everyday tasks. This paper presents the results for the final prototype regarding stability, safety and other aspects of usability.

**Methods:**

Four studies were conducted that explored the prototype in relation to the current standard frame: a detailed *gait lab study* of 9 healthy older adults performing repeated trials for a range of everyday tasks provided mechanical measures of stability, *a real-world study* that involved 9 users of walking frames provided measures of body weight transfer and lifting events, two *interview studies* (5 healthcare professionals and 7 users of walking frames) elicited stakeholder perceptions regarding stability, safety and usability.

**Results:**

Analysis of healthy older adults using a standard walking frame and the prototype frame demonstrated that the prototype increases stability during performance of complex everyday tasks (*p* < 0.05). Similarly, gait assessments of walking frame users in their home environment showed that the prototype facilitated safer usage patterns and provided greater and more continuous body weight support. Interviews with healthcare professionals and users showed that the prototype was perceived to be safe and effective and hence more usable.

**Conclusions:**

The outcomes of the separate studies all support the same conclusion: the prototype is an improvement on the status quo, the typical front-wheeled Zimmer frame for indoor use which has not changed in design for decades. The significance of this work lies in the success of the Knowledge Transfer Partnership and in biomechanics-informed design leading to improvements, which in future may be applied to other walking aids, to benefit walking aid users by promoting safer, more stable use of their aid.

## Background

As the world population is ageing [1], an increasing number of older adults are experiencing falls [2, 3]. The consequences and costs of falls are well-documented, for example, in the UK the annual cost of falls to the National Health Service is in excess of 2 billion [4]. Moreover, falls have major physical and emotional consequences [5] and in people over 75 falls are the leading cause of death [3, 4, 6]. Walking aids (e.g. rollators and walking frames) offer support during walking, yet paradoxically, walking aid use has been associated with an increased risk of falling [7–10], and to fear falling [11]. Indeed 60% of users reported problems with their walking frames, most of which were classified as “difficult and/or dangerous” [12] and other user concerns included “…could it [the walking frame] overturn when used; was it really stable?” [13].

It would appear reasonable to assume that a walking frame must be used in a stable manner to prevent a fall. Yet research has highlighted shortfalls in walking frame design that need addressing for better stability. One study explored walking aid design in relation to mechanical perturbations that reduce stability for a healthy population [14] and another reported on the centre of pressure of a rollator as a measure of balance control [15]. Inspired by the latter, Costamagna et al. developed a new method to compute the centre of pressure and the base of support for the combined user-device system to derive the stability margin of user and device [16]. The stability margin reflects how close the user-device system is to the point of “tipping over”. The approach was used in a number of studies to investigate user-device stability for a range of standard walking aids and walking tasks [16–19], one of which [18] found that users of front-wheeled Zimmer frames at times lift their device while turning, and that this is due to the wheels being fixed in-line without a swivel function. Moreover, the study reported that frames were lifted when the relatively small wheels got stuck at door thresholds or carpet edges [18]. Importantly, lifting was found to reduce user-device stability [18] as the user not only no longer receives support from their walking frame but also carries its weight. This highlighted the need for a new design that reduces the need for lifting the walking frame. Informed by these previous biomechanics studies, the authors engaged in a Knowledge Transfer Partnership in which they closely collaborated with design engineers, with the aim to develop a novel walking frame which, for the first time, is designed to better facilitate turning and crossing of thresholds without the need to lift the frame. During the course of the partnership the design process was informed by several quantitative and qualitative investigations. Findings reported in [18] led to the development of a first prototype. This first prototype was designed to reduce the need to lift the frame through 1) a set of wheels that were larger than those of standard frames to reduce the push force required to overcome thresholds, 2) through rounded ferrules attached to the rear feet of the frame to glide over thresholds where the edge of cylindrical ferrules had shown to get stuck [18], and 3) through the introduction of swivel wheels at the front of the frame to facilitate turning, but which were subject to a unique mechanism to reduce risk of the frame veering of course. A first set of gait lab and care home-based studies, involving both quantitative and qualitative methods, then assessed the first prototype in terms of mechanical stability as well as perceived usability and safety. The results of these studies led to a second prototype, for which the wheel mechanism was further tweaked and an additional set of handgrips was fitted. *This paper presents the final evaluations of the second prototype*. Specifically, the findings of four studies (two biomechanics studies conducted in the gait laboratory and the real-world, and two interview studies conducted with healthcare professionals and users of walking frames) are presented here, which together provide comprehensive insights into the success of the novel prototype design in terms of stability, safety and usability.

## Methods

Four studies (two quantitative and two qualitative) aimed to investigate the merits of the novel walking frame design. Their respective objectives were:

1. To investigate user-device stability for the new frame, as compared to a standard front-wheeled frame, in a comprehensive gait lab study of healthy older adults able to perform repeated trials.
2. To investigate surrogate measures of stability (lifting events, body weight support) for the new frame, as compared to a standard wheeled frame, in a real-world study of older walking frame users walking household distances in their home environment.
3. To investigate healthcare professionals’ perceptions with regard to the new walking frame design.
4. To investigate users’ perceptions with regard to the new walking frame design.

### The new prototype walking frame

Based on our previous work, lifting of common indoor walking frames occurred during turning, as the front wheels do not swivel and hence, to overcome floor friction, users had to lift the frame [18]. Whilst it appears an odd design choice to manufacture wheeled indoor frames which facilitate straight-line walking only, this traditional design may perhaps have originated from the safety concern that free-swivelling wheels may at times of small mediolateral imbalance make the walking frame run unintentionally sideways. Hence, for our new walking frame (the ‘prototype frame’) we developed a novel design with swivelling wheels to facilitate turning, however, the wheels are subject to a forward-bias mechanism, and thereby less likely to run unintentionally sideways. Specifically, the mechanism provides a centring force on the wheel, encouraging straight line movement. As the user attempts to turn, the centring force is overcome, thereby allowing the wheels to turn freely. When the turn is completed and the wheel orientation moves towards pointing in the direction of travel, the centring force comes into play again. Moreover, since previous work [18] had shown that lifting occurred when crossing carpet edges and door thresholds, as the standard wheels can “get stuck” on the edge of such raised surfaces, we also increased the wheel diameter by 25 mm to more easily roll across small edges as a larger wheel diameter reduces the amount of force required to push the wheel over an edge. In summary, the new prototype frame has swivel wheels which are mechanically biased to run in the forward direction, and the diameter of these wheels was increased by 25 mm.

**Fig. 1:**
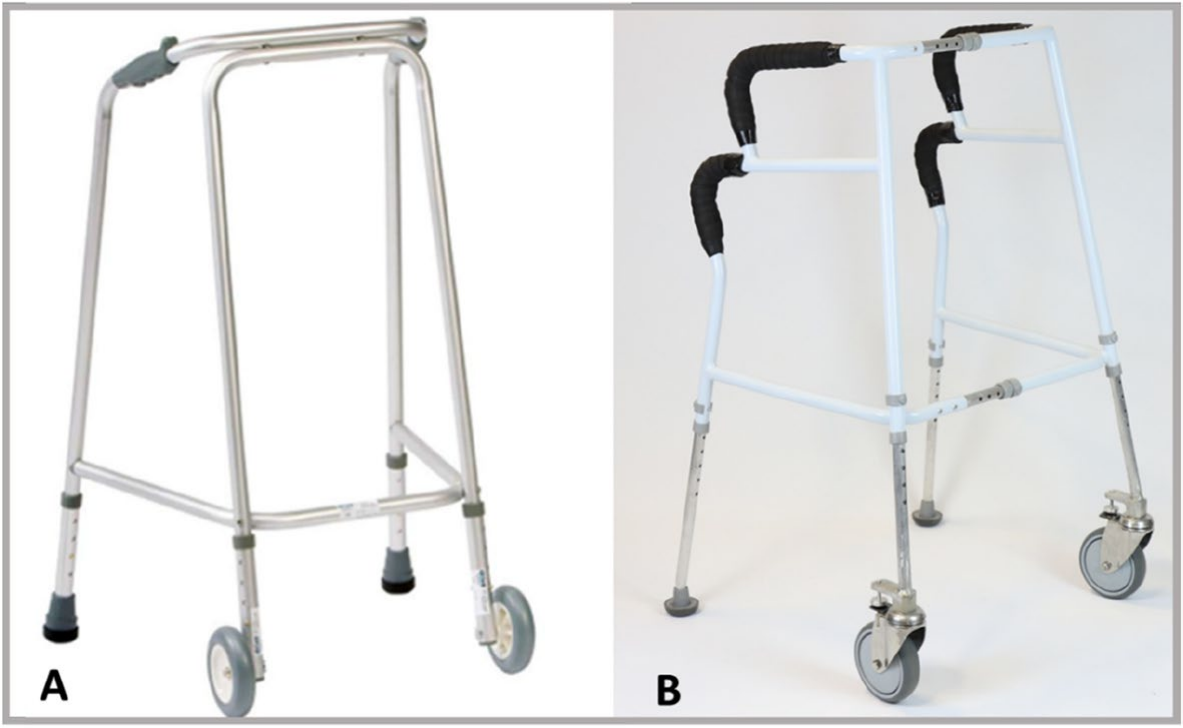
Standard frame (left) as currently prescribed and the new prototype frame (right) with additional design features

Furthermore, the prototype frame featured the following that set it apart from standard front-wheeled frames: 1) glider feet with brakes inside at the rear, that more easily glide across thresholds whilst preventing the frame from running away from its user, 2) width adjustability from 35 cm (standard width) to a maximum of 43 cm, measured from upper handle to upper handle, to accommodate users with wider hips or facilitate stability in a spacious environment, and 3) additional handgrips to support rising out of a chair and sitting down – but investigation of the various strategies with which these lower handgrips can be used goes beyond the scope of this paper which focuses on walking tasks. Figure 1 shows both the prototype frame and a standard frame as currently used in clinical practice and which served as the comparator. Notably, due to the new features the prototype frame (4 kg) was heavier than the standard frame (2 kg).

### Gait laboratory-based stability assessment

#### Participants

Nine healthy adults (3 females and 6 males, mean age ± standard deviation: 65.56 ± 4.77 years, mean body weight ± standard deviation: 77.87 ± 10.16 kg) provided informed consent and took part in the gait laboratory-based stability assessment, each providing data for walking with the standard and the prototype frame.

#### Protocol

Participants performed three tasks with both the new prototype frame and the standard walking frame: 1) without pausing, walking on vinyl flooring, crossing the edge of a carpet, and continuing to walk on carpet (‘Carpet Edge Crossing’); 2) walking in a straight line on vinyl flooring (‘Vinyl Flooring’); 3) turning 360° on carpet. Each participant performed 3 trials per task and the sequence of the tasks was random. For turning on carpet with the standard walking frame participants were instructed to 1) lift the frame at the same time as stepping (‘Turn 1’), in line with what had been observed in previous work [18], 2) lift the frame without stepping, followed by stepping when the frame was grounded again (‘Turn 2’), and 3) without stepping, attempt to drag the frame against floor friction to avoid lifting as much as possible, followed by stepping (‘Turn 3’). Since the prototype turned smoothly with the participant there were no separate movement patterns to be explored with the prototype frame. Importantly, since this paper is concerned with a comparison of the prototype frame with a standard frame, the prototype frame was kept at the standard width. We note that it was not the objective of this study to explore effects of frame width on stability, which we previously reported elsewhere [19].

#### Data collection

A 3D Motion Analysis System (Qualisys Oqus300, Qualisys AB, Göteborg, Sweden) was used to record position data of the person’s feet (by placing reflective markers on both shoes at the approximate location of the 1^st^, 2^nd^ and 5^th^ metatarsal head and the calcaneus) and of the feet/wheels of the walking frames. Regarding feet/wheels of the walking frames, a static frame trial was first recorded with clusters of 4 markers on a plate being fixated to its sides and also the front of the frame, and with a further two markers on the side of each foot/wheel of the frame. This “still shot” of the frame related walking frame feet positions to cluster marker positions, so that most of the walking frame feet markers were not needed during walking trials as they could be reconstructed from the cluster marker data [20]. Notably, for the swivel wheels of the prototype frame, markers stayed on the side of the wheel during walking trials as they did not form a rigid body with the rest of the frame. Load cells (Futek LCM300, Futek Advanced Sensor Technology Inc., Irvine, CA, USA) inside each walking frame leg recorded the four separate leg forces (in Newtons). Pressure sensing insoles (medilogic®insole, T&T medilogic Medizintechnik GmbH, Schönefeld, Germany) inside the participant’s shoes recorded the force through each of the user’s feet when in contact with the ground. The three measurement systems were temporally aligned through a sync pulse [16] and data were collected at 100Hz.

#### Data analysis

Using custom-programmed Matlab® algorithms the data were used to compute the combined stability margin of the ‘user + walking aid’ system as described in [16]. The combined stability margin is calculated from the forces through all feet in contact with the ground (both anatomical feet and walking aid feet) together with their location relative to each other (giving centre of pressure and base of support): the lower the margin, the more unstable is the ‘user + walking aid’ system. For comparison, stability values were obtained for both the prototype frame and the standard walking frame. From the stability margin trajectories, the following stability values were extracted for each participant: ‘SMmin’ which was the minimum stability margin for a given participant and walking task, and ‘Avg SMmin SS’ which was obtained by first extracting the minimum stability margin for each single support phase and then averaging these for a given participant and walking task. Stability during single support is of particular interest as, compared to double support, single support is the more vulnerable part of the gait cycle where balance may be lost. Finally, to explore to what extend the forward bias at the front swivel wheels is supporting straight line walking, the number of peaks of both frames’ mediolateral velocity trajectory were determined as a measure of side-to-side movement of each frame when walking on vinyl flooring where sliding is most likely.

Regarding further data analysis, group data for ‘SMmin’ and ‘Avg SMmin SS’ from the gait laboratory-based stability assessments did not follow a normal distribution and were hence analysed using Wilcoxon tests (for comparison of data displaying signs of skewness and kurtosis). Since the prototype frame did not require exploration of multiple movement patterns for turning, the same prototype data for turning were compared to the standard frame data for ‘Turn 1’, ‘Turn2’, and ‘Turn 3’. Count of the velocity peaks in the mediolateral direction on vinyl flooring for the standard frame and prototype frame did follow a normal distribution and corresponding data were hence analysed with a paired t-test.

### Care home gait assessment

#### Participants

In a second assessment comparing the prototype frame to the standard frame nine walking frame users (WU1-WU9, 5 females and 4 males, mean age ± standard deviation: 89.22 ± 6.40, mean body weight ± standard deviation: 71.40 ± 15.55 kg) provided informed consent and performed walking trials inside their care home.

#### Protocol

Participants performed trials such as walking in a wide communal space (‘Open Space’) where they walked in a straight line on carpet, turned through 180 degrees, and then walked back to their chair, and/or walking in a more complex area (‘Complex Space’) such as their own on-suite bedroom/bathroom area where they started their walk on carpet, crossed the door threshold into their on-suite bathroom, turned around inside the tight bathroom space, then crossed the door threshold again to walk on carpet back to their bed or chair. As for the gait lab-based study, the prototype frame was kept at the standard width for comparison with the standard frame.

#### Data collection

During their walking trials within the Open Space and Complex Space, the load cells inside the legs of the walking frames recorded force data at 100Hz.

#### Data analysis

Force data were analysed with custom-programmed Matlab® algorithms that quantified lifting events during frame use. Similar to approaches that identify toe-off from a force plate, lifts of a frame wheel/frame foot were determined as follows: first, instances where a given wheel/foot of the walking frame carried less than a quarter of that frame leg’s ‘resting load’ for 90ms or more were identified (the resting load being the load going through that wheel/foot when the frame stood on its own). A ‘Lift’ of the frame was then defined as an event where this occurred for two, three or four wheels/feet simultaneously. That outcome was then broken down further into ‘Lift all’ (all wheels and frame feet lifted), ‘Front wheels lifted’, and ‘Rear frame feet lifted’; we note that other ways to categorise lifting events are possible but as the focus was to identify if the design changes at the front and rear made a difference to lifting only these combinations were investigated separately to the more general ‘Lift’ which contains all combinations possible. Finally, the average % body weight transferred to the walking frame for both the prototype and the standard frame, as well as variability in % body weight transferred (standard deviation) were determined for each task. Variability in body weight transfer is of interest as it provides insights into the consistency with which the user receives structural support from their frame. Synchronized video in the Open Space allowed for further breakdown of the data into straight line walking and turning.

Regarding subsequent data analysis, the data from the nine care home gait assessments were then explored through figures and a table since only subsets of the nine participants walked in the Open Space and Complex Space (*n* = 7 and *n* = 5, respectively).

### Interviews with healthcare professionals

#### Participants

Five healthcare professionals (HCPs) gave informed consent and participated in the interviews: 1 occupational therapist and 4 physiotherapists (3 females and 2 males, age (mean ± SD) = 35.8 ± 7.46), with experience in the prescription of walking aids, combined working experience of 67 years and 7 months, and specialities including neurorehabilitation, community service, in-patient and geriatric therapy services.

#### Protocol

First, the new walking frame was demonstrated to the healthcare professionals and the new design features explained to them; second, the healthcare professionals tried it out next to the standard frame for a range of tasks (walking on carpet and vinyl flooring, crossing a door threshold or carpet edge, turning); and finally they participated in semi-structured interviews. Each interview began with the opening-question “What do you think of this prototype frame compared to the standard frame?”, followed by a number of general questions such as “How easy is the prototype frame to use?” and also some specific questions aimed at exploring success/failure of the new design features, for example “What do you think about turning with the prototype frame?”. Furthermore, potential barriers to clinical uptake of the prototype frame and possible strategies to overcome these were explored.

#### Data collection

Interviews were semi-structured [21, 22] and audio-recorded with a password-protected, hand-held external recording device.

#### Data analysis

All interview data were hand-transcribed and thematic analysis was used to generate suitable themes [23].

### Interviews with users of walking frames

#### Participants

Seven of the nine walking frame users (WU1, WU4-WU9) were interviewed following their walking trials with the prototype frame and standard frame (4 males & 3 females, age (mean ± SD) = 88.71 ± 6.92, body weight (mean ± SD) = 69.01 kg ± 15.19 kg).

#### Protocol

The interviews followed the same structure as for the healthcare professionals described above.

#### Data collection

As before, interviews were semi-structured [21, 22] and audio-recorded with a password-protected, hand-held external recording device.

#### Data analysis

As for the healthcare professionals’ interviews, the interview data of the walking aid users were hand-transcribed and thematically analysed [23].

### Data collation

The four studies described above differed in terms of participants, outcomes, and environment. Success of the novel frame design was therefore defined as improvement in at least one of the following outcomes compared to the standard frame design: 1) stability, 2) extent to which usage patterns were consistent with those shown to facilitate stability, i.e. reduced lifting of the frame [18]), 3) usability including aspects of effectiveness and safety as perceived by healthcare professionals, and 4) usability including aspects of effectiveness and safety as perceived by users – whilst not dropping below the standard frame’s performance in any of these.

## Results

### Gait laboratory-based stability assessment

**Fig. 2:**
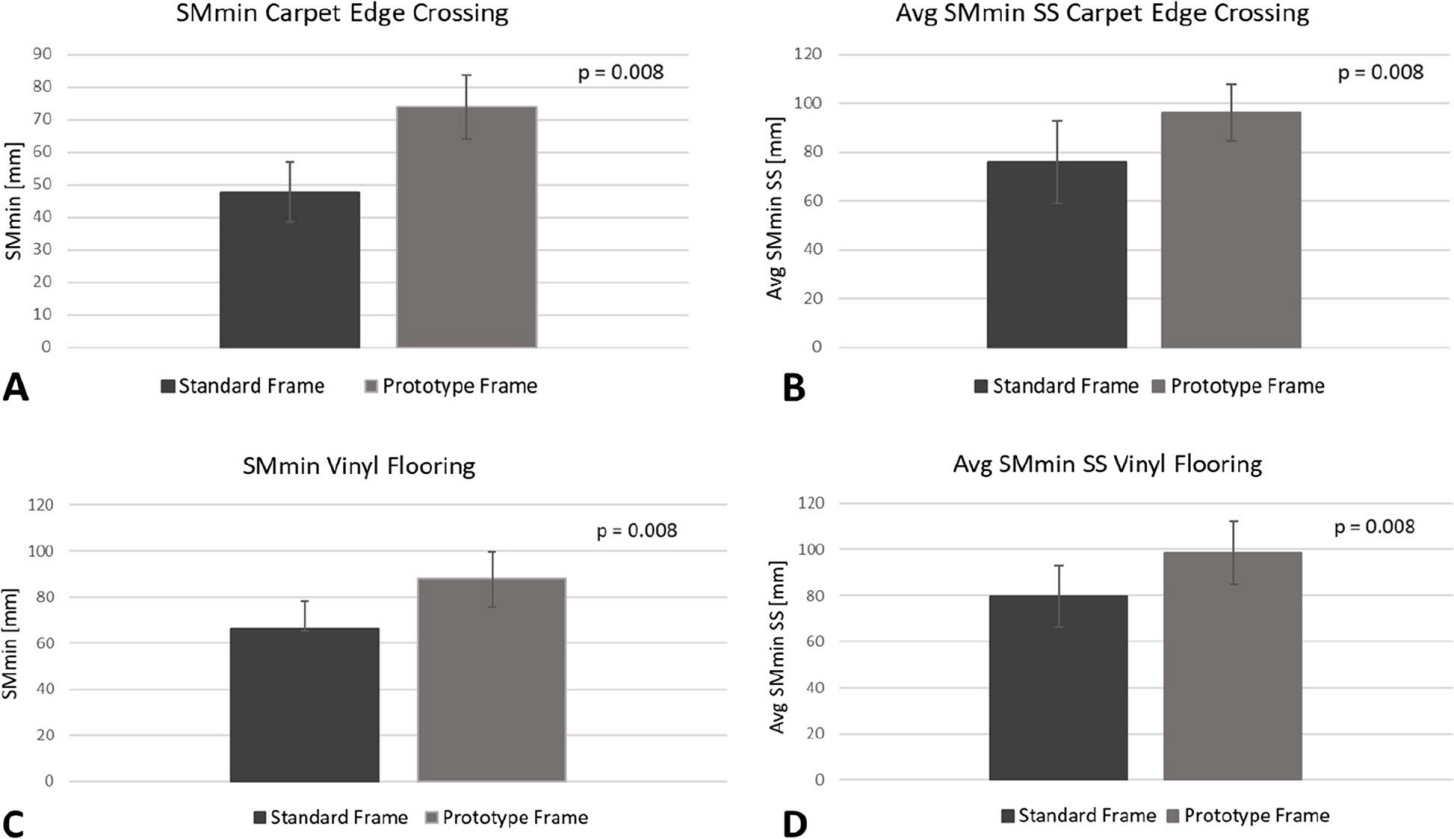
Stability results for ‘Carpet Edge Crossing’ and ‘Vinyl Flooring’. Shown are group averages of ‘SMmin’ (**A** & **C**) and ‘Avg SMmin SS’ (**B** & **D**), both in mm, together with p-values obtained from paired comparisons

Figure 2 shows the group averages of SMmin and Avg SMmin SS for the tasks ‘Carpet Edge Crossing’ and ‘Vinyl Flooring’. Stability was greater for the prototype frame as compared to the standard frame for both SMmin and Avg SMminSS (*p* < 0.01 for all).

**Fig. 3:**
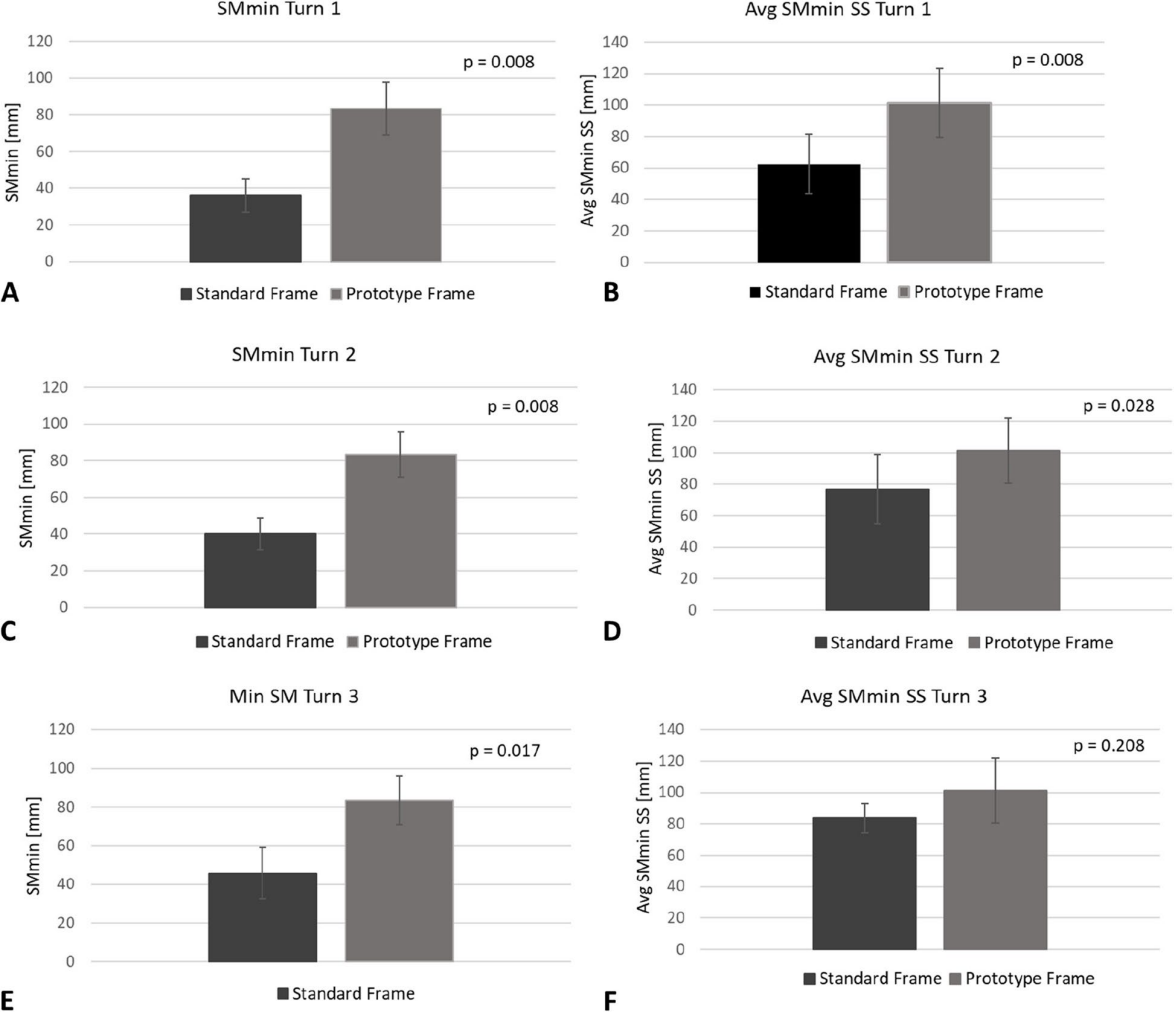
Stability results for comparison of turning with the prototype frame (only one movement pattern) to the ‘Turn 1’, ‘Turn 2’ and Turn 3’ movement patterns with the standard frame. Shown are group averages of ‘SMmin’ (**A**, **C** & **E**) and ‘Avg SMmin SS’ (**B**, **D** & **F**), both in mm, together with p-values obtained from paired comparisons

Figure 3 shows the group averages of SMmin and Avg SMmin SS for the comparison of turning with the prototype frame to the ‘Turn 1’, ‘Turn 2’ and Turn 3’ movement patterns of the standard frame. Stability was greater for the prototype frame as compared to the standard frame, with 3 comparisons with p values less than 0.01, 2 comparisons with p values less than 0.05 and one comparison with a p value of 0.208.

Investigation of the number of peaks in both frame’s mediolateral velocity profile for walking on vinyl flooring showed a slightly greater number of peaks for the prototype frame than the standard frame (mean ± SD = 34 ± 8 for the standard frame and 39 ± 11 for the prototype frame; *p* > 0.05).

### Care home gait assessment

**Fig. 4:**
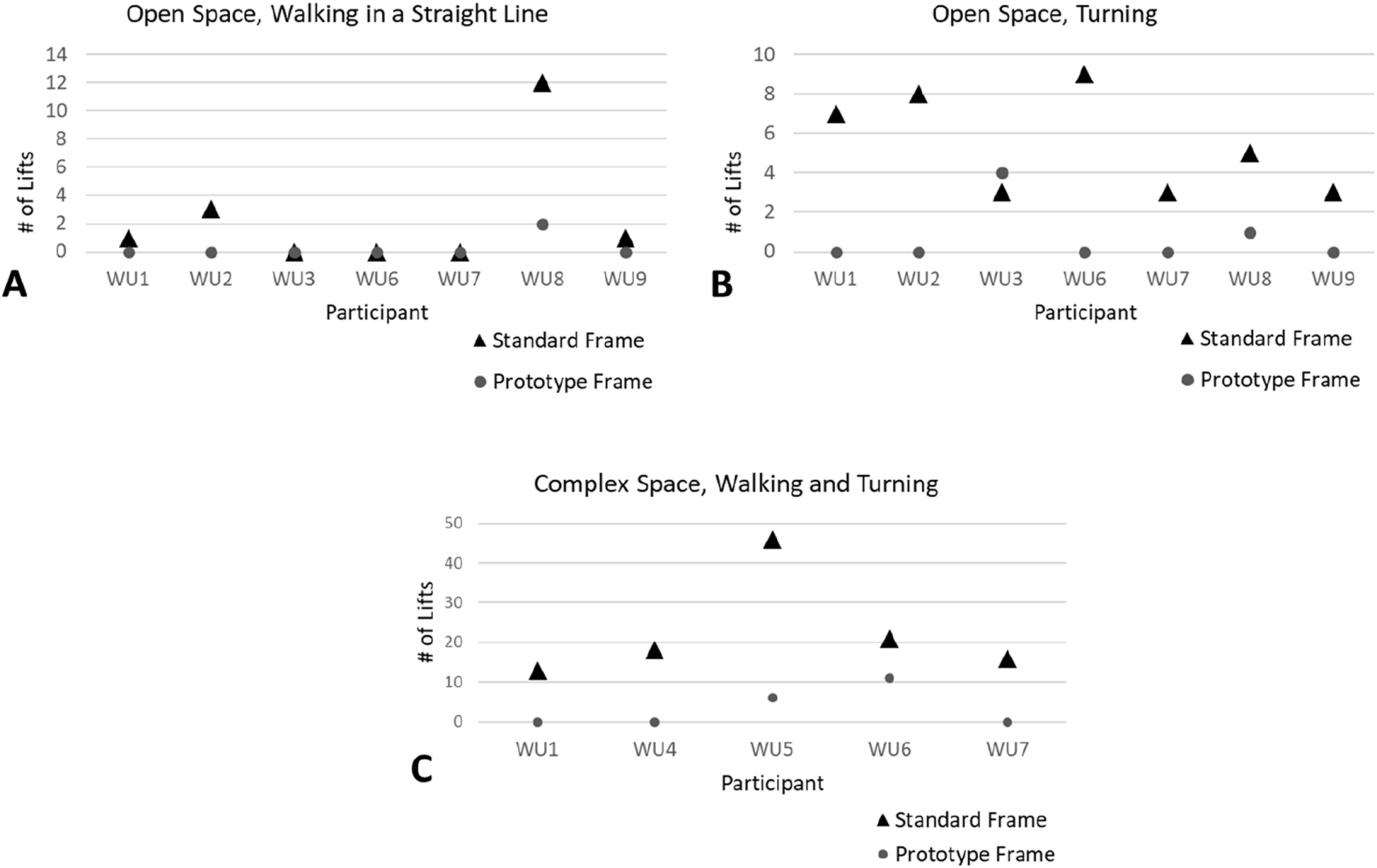
Lifting events (less than 3 frame legs are grounded). Individuals’ data (WU1-WU9) for the standard frame and the prototype frame are shown for walking in a straight line in the Open Space (**A**), turning in the Open Space (**B**), and walking in combination with turning in the Complex Space (**C**)

Regardless of walking condition (Open Space, Complex Space), the number of general ‘Lifts’ for the group of frame users was smaller for walking with the prototype frame as compared to the standard frame (Fig. 4). Specifically, in 15 cases shown across Fig. 4 A-C the prototype frame reduced the number of ‘Lifts’, in 3 cases ‘Lifts’ were zero for both frames, and only in one case (participant WU3 turning in the Open Space) was one additional ‘Lift’ observed for the prototype frame.

Investigation of the subset of special lifting events revealed 31 ‘All wheels and feet lifted’ events, 60 ‘Front wheels lifted’ events, and 70 ‘Rear feet lifted’ events for the standard frame, whilst the prototype frame was lifted only 9 times at the front and once at the rear (Table 1).

**Table 1:** Special lifting events “All legs lifted”, “Front legs (wheels) lifted” and rear legs lifted for individuals WU1-WU9 walking in the Open Space and/or Complex Space with the standard frame and prototype frame

	**All legs lifted**		**Front wheels lifted**		**Rear feet lifted**	
	**Standard frame**	**Prototype frame**	**Standard frame**	**Prototype frame**	**Standard frame**	**Prototype frame**
**Open Space**						
**WU1**	6	0	7	0	6	0
**WU2**	1	0	4	0	6	0
**WU3**	0	0	3	0	1	0
**WU6**	5	0	3	0	1	0
**WU7**	0	0	2	0	6	0
**WU8**	6	0	3	0	10	0
**WU9**	1	0	2	0	1	0
**Complex Space**						
**WU1**	3	0	11	0	8	0
**WU4**	0	0	3	0	1	0
**WU5**	7	0	11	4	22	0
**WU6**	0	0	6	5	3	1
**WU7**	2	0	5	0	5	0
**TOTAL**	31	**0**	**60**	**9**	**70**	**1**

**Fig. 5:**
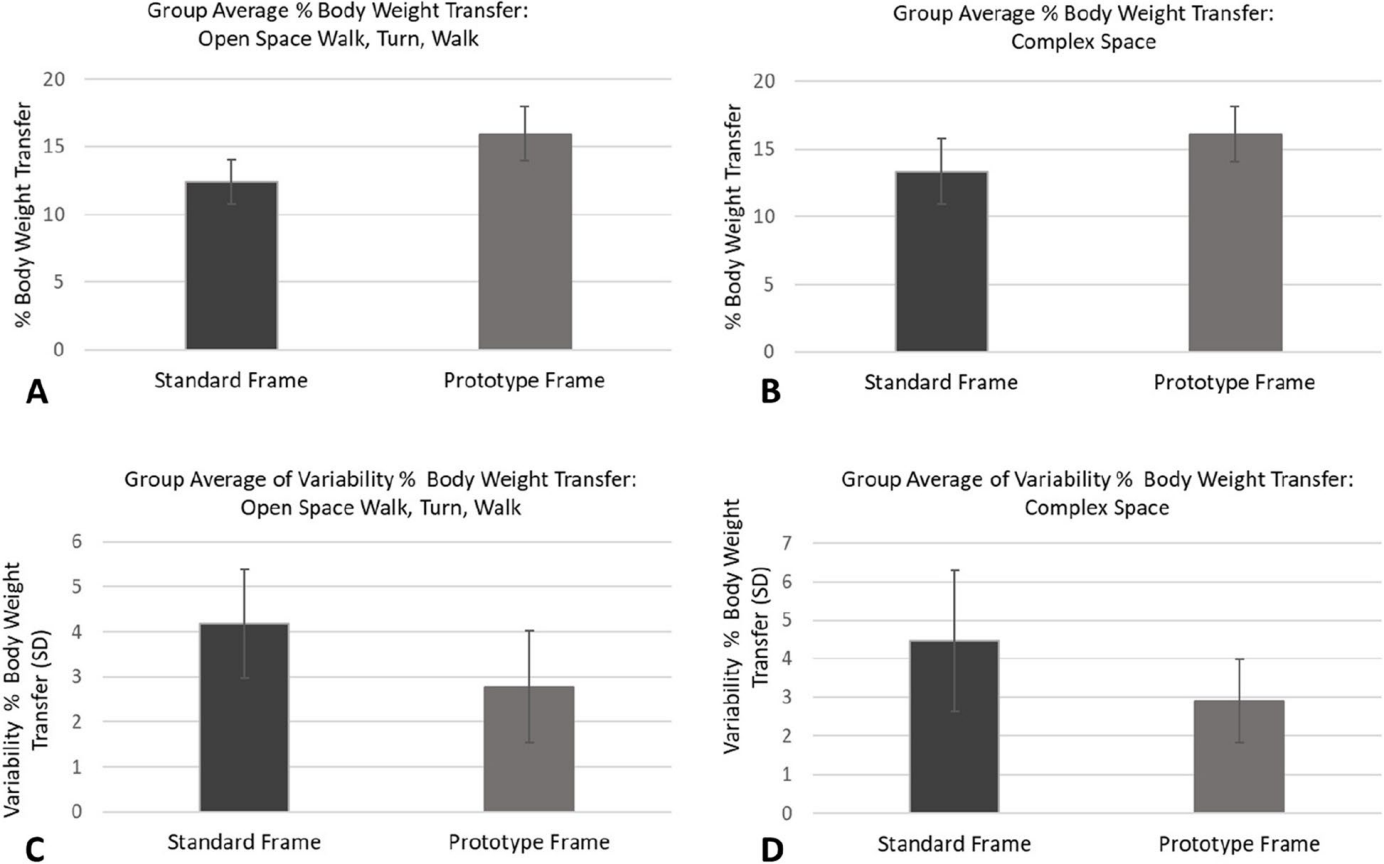
Group averages for % body weight transfer and variability in % body weight transfer for individuals walking and turning in the Open Space (**A** & **C** respectively; participants WU1, WU2, WU3, WU6, WU7, WU8, WU9) and Complex Space (**B** & **D** respectively, participants WU1, WU4, WU5, WU6, WU7). Error bars indicate standard deviation of the respective group data

At the same time, the prototype frame increased the average % body weight transferred to the frame (Fig. 5 A & B) whilst reducing variability in % body weight transferred (Fig. 5 C & D).

### Interviews with healthcare professionals

Three themes emerged from the thematic analysis of the interviews with healthcare professionals: 1) Usability, 2) Dimensions and Weight, 3) Barriers to Clinical Uptake.

#### Usability

Usability of a device can be described as the ability to use it for task performance in a safe, effective and efficient manner whilst enjoying the experience [24]. A number of comments were made by the healthcare professionals (HCP1-HCP5) that highlighted how the different features of the prototype frame make it safe, effective, efficient, and that users may enjoy it:

*Safety*: All five HCPs felt that the frame was generally safe and secure:

“Yeah, it feels safe and secure doesn’t it?” (HCP1)

“It feels perfectly secure. Certainly, more than the traditional one.” (HCP2)

“I think so {it is safe and secure}, I think if you think of it just as a replacement for the standard wheeled Zimmer frames that we have now.” (HCP3)

“I think, the turning circle has been reduced in comparison to the standard frame and I think that it improves safety because of this, because you can turn in a smaller space. You are not having to manoeuvre the patient into unsafe positions that is a very good point on the turning, and I also think that because of the turning circle you are stopping from lifting and again reducing risks for falls and it increases stability” (HCP4)

“I do prefer this frame {referring to the prototype frame when asked about feeling safe and secure}” (HCP5)

However, two HCPs had some reservations in terms of safety for particular patients such as those with cognitively impairment, for example: *“My biggest worry about stability and security is patients with cognitive impairments.”* (HCP4).

*Effectiveness & Efficiency*: All five HCPs made positive comments on the various features of the prototype frame and how these facilitate effective and efficient use of the frame:

“Yeah. It went through quite smoothly off the carpet strip {door threshold}, sorry, flowed nicely… The glider feet went over nicely, and the wheels seem to follow ok as well…it comes around nicely as well, doesn’t it … I think the wheels at the front {I like best} because they can change direction…It is, yeah {the forward bias mechanism is working}.” (HC1)

“I would say the definite advantage is I can see are the wheels. So that ability to not have to ask someone to statically sort of or incrementally turn around could have major benefits and that ease of getting over the threshold from one surface to another… I think again it makes it easier because you got the larger wheel to get over {the carpet edge}… I tried it from all different angles and it seemed to get there. No problem at all.” (HC2)

“I definitely like the wheels that are slightly more like the kitchen trolley… I think I like the wheels even though with a bit of tweaking {of the forward bias} I would like them more… I thought it went up {the door threshold} ok actually… I think they allowed a bit of a brake {the rear legs}, you know they provided some brake to it so it wouldn’t run off with you as a trolley would.” (HC3)

“I would rather have the newer frame because I can turn, it helps me stand and it gives me a stronger base of support throughout all movements, it won’t fall away from you. So, yes, I prefer the new frame over the {standard} frame.” (HC4)

“It is very helpful comparatively for the {standard} one…Yeah, turning is definitely better with the new frame…Yeah, that is so smooth and very easy to move…” (HC5)

*Enjoyment*:“*I mean, the front is very much like a kitchen trolley and everybody loves a kitchen trolley*.” (HC1)

Notably three HCPs immediately expressed a clear preference for the prototype frame whilst two were not able to say which they prefer on the day, as they felt they need to try it with patients first.

#### Dimensions & weight

All five HCPs found the weight of the prototype frame acceptable, with two HCPs identifying the extra weight as a benefit as it may discourage lifting and make the frame sturdier:

“No {the weight is not a problem}. I think for some patients they might find it more reassuring because they do find them {standard frames) quite a light weight” (HCP1) and “For me I can say that absolutely the weight is benefits for us…Stops lifting…if we have heavy things…we will feel more stability and comfortable.” (HCP5).

Moreover, four HCPs liked the width adjustability, for example: *“I think the changing width is really nice… because if somebody has not got much space in the house, could make it a bit smaller, someone with a larger person, then you don’t need to then special order a narrow frame or a wide frame, you’ve just got a frame that fits more people. So, I think that’s really a nice feature.”* (HCP3) and *“Yeah, the idea that width is adjustable is really useful”* (HCP4).

#### Barriers to clinical uptake

Winning therapists over and convincing commissioners of the extra cost were seen as barriers to uptake. To overcome the former, it was suggested to get “champions” from relevant physiotherapy organizations and groups and to publish in the professional magazines they read. It was also suggested to demonstrate benefits against extra cost through further work.

### Interviews with users of walking frames

Three themes emerged from the thematic analysis of the interviews with users (WU1 and WU4-9):

#### Usability

As for the healthcare professionals, comments on usability spanned aspects of safety, effectiveness, efficiency and enjoyment:

*Safety:* All seven of the participants said that they felt safe with the prototype frame:

“Yes, I did {feel safe}.” (WU1)

“I didn’t feel I was going to fall over or make any fall. Just went nice and steady” (WU4)

“This is top marks for me at the moment {in terms of feeling safe}” (WU5)

“Yes, I felt safe with it, yes. And yes it felt firm. Firm as in {with} my own {standard frame} which sort of, you soon knock that {standard frame} over, wouldn’t you, you wouldn’t {knock over} this {prototype frame}, no, no. It feels more secure. Yeah.” (WU6)

“I would say it was safe.” (WU7)

“Quite, quite happy with it {in terms of feeling safe and secure}” (WU8)

“Yes, I felt safe enough.” (WU9).

However, WU9 did express that they felt they needed to be a “*more careful*” with it “*Because it could run away more easily*.”.

*Effectiveness & Efficiency:* Every participant made positive comments that reflected effectiveness of the prototype frame in supporting performance of tasks and the efficiency with which tasks could be performed, for example.

“It did what it did and it was what I expected it to do and it did it.” (WU1)

“That’s better than what I am using at the moment.” (WU4)

“Much better. Much better. Far much better. Better obviously with the swivel wheels.” (WU5)

“It {turning} is very easy. Yes. Yes. Oh, very, because normally you have to pick it up and plonk it down. This {prototype frame} I think is superior. Yes, I think this is far superior for going around here.” (WU6)

“I think probably the feet and these wheels that turn has set me an advantage.” (P7)

“Yes, they (the swivel wheels) were very good. I liked it.” (WU8)

*“*No problem {crossing the door threshold}, that was easy.” (WU9)

Notably, six participants (WU4-9) made explicit comments that the prototype frame was easy to use, and the remaining participants didn’t think that there was anything they *“found any more difficult or easy”*, they *“just used it”* (WU1). Comments were therefore predominately positive with four users expressing a clear preference for the prototype frame, however, three participants did remark that it takes some getting used to the prototype frame. Moreover, one user voiced need for further refinement of the way in which the rear brakes come on, and another commented on the frame ‘sliding’ on vinyl flooring.

*Enjoyment:* Some comments were made that demonstrated clear enjoyment of using the prototype frame:

“I enjoyed using this one {prototype frame} compared to the other {standard frame}.” (WU4)

“I enjoyed using it {prototype frame}, yes.” (WU6)”

“You can leave it {with me} if you like…” (WU8)

#### Dimensions & weight

Overall participants were happy with the dimensions and weight of the prototype frame for their everyday use, stating that dimensions were *“alright”* (WU1) or *“OK”* (WU5, WU7, WU9) and WU8 and WU9 both stated that they didn’t notice any difference in weight. The extra weight due to the front wheel design was considered to be stability enhancing by one participant: *“It is a good thing {the additional weight}. Yes. It feels more firm, if you did stumble sort of thing, yes, yes."* (WU6). However, two comments were made that the extra weight may impact on getting it into the boot of a car *“I can't imagine this {prototype frame} going into the boot of a car."* (WU6) and *“But I suspect, the weight of it is going to get more of a problem to me… because my friends are as old as me, it can be less and less likely to take me out” {if too heavy for them to lift the frame in the boot}”* (WU7).

#### Cost & colour

WU1 and WU9 felt the prototype frame should cost about the same as the standard frame, but the other five felt it can cost more (as much as £100 for two of them). Interestingly, only three participants felt that a choice in colour would be desirable, and two preferred the standard frame’s silver-grey whilst two others had no preference.

## Discussion

The research project reported here included four studies, which together provide a comprehensive evaluation of the prototype frame design. The approach taken is in line with recent research that likewise used objective and subjective measures for evaluation of an intelligent walker [25], however, the prototype frame evaluated here is a passive device and its novel features do not require power. Quantitative stability analysis of healthy older adults using a standard walking frame and the prototype frame demonstrated that the prototype frame design increases stability during performance of complex everyday tasks: the stability margin improved by ~ 10–40 mm in some cases indicating that the user-device system was further from the point of “tipping over” as compared to the standard frame. This outcome is positive considering that a decreased margin of stability in unassisted walking has been associated with a history of falls [26]. Moreover, we are encouraged by a recently published study which reported the circumstances of falls among older adult walker users in long-term care [27]. They reported a link between poor manoeuvrability and lateral stability of the two-wheeled walking frames, and falling, and identified a need for device improvements [27]. Similarly, gait assessments of walking frame users in their home environment showed that the prototype frame facilitated safer usage patterns and provided greater and steadier (less variable) body weight support for improved stability; latter is particular important considering that loss of support has been identified as a cause of falls in frame users [28]. Finally, interviews with healthcare professionals and users of walking frames provided supporting evidence regarding the frame’s usability in terms of safety, effectiveness, efficiency, and enjoyment. The findings of the 4 studies show that the prototype frame demonstrated improvements over the standard frame in all four outcome areas (stability, extent of safe usage patterns, and usability from both the clinicians’ and users’ perspectives). To the best of our knowledge, to date no walking aid has been investigated in such detail, with current ISO standards focusing on structural integrity of the device without considering user-device interaction (as described in Section 6.6, 15.2.2, 15.2.3, 15.2.4 of ISO 11199–1: 2021). We note that this paper presents the findings for the final prototype; another had previously been investigated in the same way and served to inform further refinements that led to the design reported on here. Hence the evolution of this design has been underpinned by repeated quantitative analyses and stakeholder feedback.

Whilst the sample sizes for the individual studies may be considered small, it is encouraging that the outcomes of the separate studies all point in the same direction, namely that the design is an improvement on the status quo, the typical front-wheeled Zimmer frame for indoor use which has not changed in design for decades. Notably, the design of the forward bias mechanism was successful; the prototype frame with its swivel wheels only slightly increased side-to-side movement during walking on vinyl flooring, yet this was not significant at the 0.05 level and stability was not compromised. This work demonstrates how knowledge transfer from academia to industry can inform the design of better, safer walking aids that have the potential to promote active aging and are hence fit for twenty-first century living. Indeed, the 2005 review by Bateni and Maki concluded that more research on walking aids is needed, which could lead to improved walking aid design for safer and more effective use [29]. This has inspired our research journey over the years leading to the industry collaboration reported here.

As discussed in the Background, the prototype frame has also been designed to support rising from a chair and sitting down again. The use of the second set of hand grips to support the user in standing up and sitting down is still being investigated in a new project, and hence this paper focuses only on walking with the frame. Considering recent findings concerned with use of handgrips on a simulated rollator frame that showed to be beneficial in a study of healthy adults rising up and sitting down [30], analysis of these transfers merits further study.

A few papers stand out that previously reported on walking frame design and/or assessed the biomechanics of walking aid use:

In 2004 Bateni et al. suggested raising the lower cross bar to enable compensatory stepping in the presence of mediolateral perturbations [14]. Our design did not adopt that feature as there was no real-world evidence on the frequency with which mediolateral compensatory steps may occur in actual users (such events were not observed in [18]), and at the same time it would weaken the frame structure and may risk failing the required ISO tests that the frame has to pass.

A key aspect of walking frames is that they are typically used by frail individuals, many of whom may not be willing or able to visit a university gait laboratory, but whose interactions with their walking aids need to be understood if we are to advance the design of walking aids. One of the few studies to have previously addressed this issue is Tung et al. who reported on outcome measures reflective of balance control using both real-world and gait lab-based assessments of rollator users [15]. Inspired by their approach we also collected data in the lab as well as the home environment. Our study involved more participants than Tung’s, and complemented the biomechanical measures with qualitative interviews of users and healthcare professionals.

Last but not least, recent research has seen development of intelligent walking aids, for example the aforementioned intelligent walker [25] and also a rollator that detects obstacles and facilitates avoidance of collisions [31]. The prototype frame discussed here may also benefit from such features, however, research would be needed to identify whether reliable recharging is feasible in the walking aid user population, how users with cognitive challenges might interact with such a device and also the cost implications for purchasers. It is worth noting that the manufacturing costs of our prototype device are expected to be in line with lower-end rollators that are widely prescribed. Notably, research has also shown that user training has the potential to enhance effective gait aid use [32], and hence development of appropriate guidance materials for the new frame is needed.

## Conclusions

The work was undertaken as part of a Knowledge Transfer Partnership between academic biomechanists and industrial design engineers employed by a walking aid manufacturer. A series of quantitative and qualitative studies informed the design, with the final evaluation of the second prototype frame presented here. Rigorous biomechanical and qualitative evaluations of the prototype frame demonstrated clear advantages over the current standard frame across each of four studies, hence supporting the idea that the design facilitates more stable walking than the standard front-wheeled frame, and is perceived to be both safe and effective. The significance of this work lies in successful knowledge transfer leading to an improved walking frame design, an approach which in future may facilitate other walking aid designs being informed by biomechanical evidence and stakeholder feedback. The timeliness lies in the growing prevalence of frailty in our ageing population [33] and the reported increase in users of walking frames over time [34]. A longitudinal study is now needed to assess the performance of users with the new frame in everyday life, including the impact of the new frame on falls incidence and mobility.

## Data Availability

The datasets generated and/or analysed during the current study are not publicly available due to the industry investment in the project and associated collaboration agreement which states that all IP generated remain with the company partner. However, data can be made available from the corresponding author on reasonable request and with permission of NRS Healthcare.
